# Millimeter Wave Treatment Inhibits Apoptosis of Chondrocytes via Regulation Dynamic Equilibrium of Intracellular Free Ca^**2+**^

**DOI:** 10.1155/2015/464161

**Published:** 2015-01-29

**Authors:** Jinxia Ye, Guangwen Wu, Xihai Li, Zuanfang Li, Chunsong Zheng, Xianxiang Liu, Hongzhi Ye

**Affiliations:** ^1^Academy of Integrative Medicine, Fujian University of Traditional Chinese Medicine, 1 Qiuyang Road, Minhou Shangjie, Fuzhou, Fujian 350122, China; ^2^Academy of Integrative Medicine Biomedical Research Center, Fujian University of Traditional Chinese Medicine, 1 Qiuyang Road, Minhou Shangjie, Fuzhou, Fujian 350122, China; ^3^Fujian Provincial Key Laboratory of Integrative Medicine on Geriatrics, Fujian University of Traditional Chinese Medicine, 1 Qiuyang Road, Minhou Shangjie, Fuzhou, Fujian 350122, China

## Abstract

The molecular mechanisms of TNF-*α*-induced apoptosis of chondrocyte and the role of Ca^2+^ mediating the effects of MW on TNF-*α*-induced apoptosis of chondrocytes remained unclear. In this study, we investigated the molecular mechanism underlying inhibiting TNF-*α*-induced chondrocytes apoptosis of MW. MTT assay, DAPI, and flow cytometry demonstrated that MW significantly increased cell activity and inhibited chromatin condensation accompanying the loss of plasma membrane asymmetry and the collapse of mitochondrial membrane potential. Our results also indicated that MW reduced the elevation of [Ca^2+^]*_i_* in chondrocytes by LSCM. Moreover, MW suppressed the protein levels of calpain, Bax, cytochrome c, and caspase-3, while the expressions of Bcl-2, collagen II, and aggrecan were increased. Our evidences indicated that MW treatment inhibited the apoptosis of chondrocytes through depression of [Ca^2+^]*_i_*. It also inhibited calpain activation, which mediated Bax cleavage and cytochrome c release and initiated the apoptotic execution phase. In addition, MW treatment increased the expression of collagen II and aggrecan of chondrocytes.

## 1. Introduction

Osteoarthritis (OA) is characterized by a progressive loss of joint articular cartilage caused by the interaction of mechanical and biological factors. The abrasion of articular cartilages stimulates an inflammatory reaction in synovial cells and chondrocytes. The local increase in inflammatory stimuli in the articular tissues leads to secondary synovial proliferation, articular exudation, and cartilage dystrophy, resulting in articular cartilage degeneration [1–4].

One of the pivotal inflammatory factors, tumor necrosis factor-*α* (TNF-*α*), induces inflammation, promotes the release of bone matrix-degrading enzymes, and mediates fibroblastic hyperplasia and histological damage [5, 6]. Depending on the cell type and the state of cellular activation, TNF-*α* can induce a wide range of biological effects, including cell differentiation, proliferation, apoptosis, and multiple proinflammatory effects [7].

Millimeter wave is a high-frequency electromagnetic wave with a wavelength of 1~10 mm and a frequency of 30–300 GHz. As it has been shown to produce multiple biological effects, MW is beneficial in the treatment of OA. Living organisms generate 0.5 × 10^10^~3 × 10^12^ coherent oscillations during metabolism, which is within the frequency of a millimeter wave. Therefore, the energy of the MW can be absorbed by biological tissues through resonance, and the energy may interfere with signal transduction and affect metabolism [8–12].

Our preliminary work demonstrated that MW inhibits the activation of the TNF-*α*-mediated NF-*κ*B signal transduction pathway [13]. To further discover the millimeter wave's mechanism of action, we used TNF-*α* to induce apoptosis. Calcium is one of the most important secondary messengers in cells, and so it plays an important role in apoptosis. Free Ca^2+^ and its binding proteins are important and conserved components of intracellular signaling networks [14, 15]. In this study, we measured the change in concentration of intracellular free calcium ([Ca^2+^]_*i*_) by dyeing chondrocytes with fluo-3/AM, exposing them to MW, and observing the change of [Ca^2+^]_*i*_ with a laser scanning confocal microscope (LSCM).

## 2. Materials and Methods

### 2.1. Instruments and Equipment

KFA-100A millimeter wave therapeutics at wavelengths of 7.5–10.0 mm, a power density of 4 mw/cm^2^, and a radiation area of 15.1 cm^2^ (45.6 ± 4 mm long and 33.2 ± 3 mm wide) were manufactured by Zhongcheng Kangfu Technology Co., Ltd. (Beijing, China). A confocal laser scanning microscope (LSM 710) was obtained from Carl Zeiss (Göttingen, Germany). A fluorescence-activated cell sorting (FACS) calibur (FACSCalibur) was obtained from Becton, Dickinson and Company (San Jose, California).

### 2.2. Reagents

Fetal bovine serum (FBS), Dulbecco's modified Eagle's medium (DMEM), and trypsin were purchased from HyClone Laboratories, Inc. (Logan, Utah). Type II collagenase, TNF-*α*, fluo-3/AM, and a 3-(4,5-dimethylthiazol-2-yl)-2, 5-diphenyltetrazolium bromide (MTT) colorimetric assay were obtained from Sigma-Aldrich Corporation (St. Louis, Missouri). Toluidine blue was obtained from Guoyao Group (Beijing, China), and a type II collagen immunostaining kit from Boster Inc. (Wuhan, China). An apoptosis assay, the Annexin V-FITC Apoptosis Detection Kit II, was provided by Becton, Dickinson and Company (San Jose, California). A JC-1 mitochondrial membrane potential detection assay was obtained from Invitrogen (Grand Island, New York). Hoechst 33258 was provided by the Beyotime Institute of Biotechnology (Nantong, China). The antibodies calpain, Bax, Bcl-2, cytochrome c, caspase-3, collagen II, and aggrecan were obtained from Beijing Boao Sen Biotechnology Co., Ltd. (Beijing, China). An HRP secondary rabbit anti-goat antibody was obtained from Zhongshan Golden Bridge Biotechnology Company (Beijing, China).

### 2.3. Animals

Thirty 4-week-old specific pathogen-free (SPF) male Sprague Dawley (SD) rats were purchased from Shanghai SLAC Laboratory Animal Center Inc. (Shanghai, China) and raised in a sterile environment. The Laboratory Animal Use Certificate number for these animals is SCXK(SH)2007-0005. The treatment of the laboratory animals in this study complied with* Guidance Suggestions for the Care and Use of Laboratory Animals Welfare*, published by the Ministry of Science and Technology of China [16].

### 2.4. In Vitro Culture of Rat Chondrocytes

The rat chondrocytes were isolated and cultured as previously described [12]. The cells were counted with a blood cell count plate to adjust the concentration of the cell suspension to 2-3 × 10^5^ mL. The cells were seeded in flasks and cultured at 37°C in a 5% CO_2_ incubator [17].

### 2.5. Identification of Chondrocytes Using Toluidine Blue Staining and Immunohistochemical Staining for Type II Collagen

The third generation chondrocytes were seeded on cover slips and cultured for 72 h. The cells were washed with PBS, fixed in 4% neutral formalin for 30 min, and stained with 1% toluidine blue for 30 min [12]. The cover slips were rapidly washed in ethanol, dried, and mounted for microscopic examination. The expression of type II collagen was observed using immunohistochemical staining [18]. Images were captured at a magnification of ×20.

### 2.6. Millimeter Wave Stimulation

The millimeter wave detecting head was positioned 30 mm above the cultured cells in the MW treatment group. The chondrocytes were placed around the probe within a 20 mm radius and within the radiation area of (33.2 ± 3) × (45.6 ± 4) mm [19]. Following millimeter wave treatment, the apoptotic rate of the chondrocytes was examined by flow cytometry (FCM) using Annexin V-FITC/PI and the mitochondrial membrane potential (ΔΨ*m*) by FCM analysis with JC-1 staining. The fluorescence intensity of the chondrocytes loaded with fluo-3/AM was detected by measuring the intracellular free Ca^2+^ with an LSCM with 488 nm excitation and 525 nm emission wavelengths. The protein expression levels of calpain, Bax, Bcl-2, cytochrome c, and caspase-3 were measured by Western blot.

### 2.7. Cell Treatment

The third-passage chondrocytes were seeded into 6-well plates at a density of 1 × 10^5^/mL in 2 mL of medium for 72 hours. The cells were subsequently divided into 3 groups: a control group (normal culture without treatment), a 10 ng/mL TNF-*α* group (treatment with 10 ng/mL TNF-*α* for 8 hours and no MW signal), and a 10 ng/mL TNF-*α* + MW 30 group (treatment with 10 ng/mL TNF-*α* for 8 hours and MW signal for 30 minutes).

### 2.8. Evaluation of Cell Viability by MTT Assay

The activity of the chondrocytes was examined with the 3-(4,5-dimethylthiazol-2-yl)-2, 5-diphenyltetrazolium bromide (MTT) colorimetric assay. Third-passage chondrocytes were seeded into 96-well plates at a density of 1 × 10^5^/mL in 0.1 mL of medium. The cells were equally and randomly divided into 3 groups of 6 wells each, which were supplemented with 10% FBS in DMEM at TNF-*α* final concentrations of 10 ng/mL for 8 hours. The cells in the MW group were inverted by millimeter wave for 1 hour. At the end of the treatment, 100 *µ*L MTT (0.5 mg/mL in phosphate-buffered saline [PBS]) was added to each well, and the samples were incubated for an additional 4 hours at 37°C. The purple-blue MTT formazan precipitate was dissolved in 100 *µ*L dimethyl sulfoxide (DMSO). The absorbance was measured at 490 nm using an ELx808 absorbance microplate reader (BioTek Instruments Inc., Vermont). The relative cell viability was expressed as the ratio (%) of the absorbance in the experimental wells to that of the control wells.

### 2.9. Intracellular [Ca^2+^]_*i*_ Measurements

Chondrocytes were seeded into special plates of the LSCM at a density of 1 × 10^5^/mL in 2 mL of medium for 72 hours. Following 4 hours of treatment with 20 ng/mL TNF-*α*, the chondrocytes were washed 3 times with PBS. The cells were then loaded with 5 *μ*mol/L fluo-3 AM of Ca^2+^ indicator and 10 *μ*mol/L Hoechst 33258 in final volume in the dark at 37°C incubator for 1 hour. The fluorescence signals of all groups were detected and recorded with the LSCM at 488 nm excitation and 525 nm emission wavelengths. The cells of the MW group were treated by millimeter wave.

To detect the fluorescence intensity of the intracellular Ca^2+^ imaging, five visual fields for each group were randomly selected and five cells for each visual field were observed and analyzed with the LSCM for 30 minutes. The parameters of illumination and detection were digitally controlled to maintain the same settings throughout the experiments [20].

### 2.10. DAPI Staining of Apoptotic Chondrocytes

The treated chondrocytes were collected and fixed in 4% paraformaldehyde for 5 minutes. The fixation solution was discarded and the cells were rinsed 3 times in 1x PBS. The buffer was discarded and the cells were incubated in 10 *μ*M DAPI solution in the dark at 37°C for 15 minutes. The DAPI solution was removed and the stained cells were rinsed 3 times in 1x PBS and supplemented with fresh 1x PBS buffer. The stained cells were examined for cellular morphology and nuclear profiles under an inverted fluorescent microscope. Ten visual fields in each group were randomly selected and the number of apoptotic chondrocytes in each of these fields was counted.

### 2.11. Annexin V-FITC/PI Assay of Apoptotic Rate of Chondrocytes

Following treatment with TNF-*α* and MW, chondrocyte apoptosis was determined by FCM using the FACSCalibur and the Annexin V-FITC/PI Apoptosis Detection Kit II. Staining was performed according to the manufacturer's instructions, as previously described [21]. Four cell populations were identified according to the following interpretations: viable population in the lower-left quadrant (PI-negativity and FITC-negativity signals), early apoptotic population in the lower-right quadrant (PI-negativity and FITC-positivity signals), necrotic population in the upper-left quadrant (PI-positivity and FITC-negativity signals), and late apoptotic or necrotic population in the upper-right quadrant (PI-positivity and FITC-positivity signals) [19].

### 2.12. Measurement of Mitochondrial Membrane Potential (ΔΨ*m*) by Flow Cytometry Analysis with JC-1 Staining

The collapse of an electrochemical gradient across the mitochondrial membrane during apoptosis was measured using a JC-1 mitochondrial membrane potential detection kit by FCM. This kit uses a unique cationic dye, JC-1, to signal the loss of the mitochondrial membrane potential. In healthy cells, the dye accumulates in the mitochondria as aggregates, which become fluorescent red. In apoptotic cells the mitochondrial potential collapses, and the JC-1 cannot accumulate within the mitochondria and remains in the cytoplasm in a green fluorescent monomeric form. These different forms of JC-1 were detected by FCM as described by the manufacturer [21].

### 2.13. Western Blot Analysis

Chondrocytes were seeded into 25 cm^2^ culture flasks at a density of 1 × 10^5^/mL in 4 mL of medium for 72 hours. Following treatment with TNF-*α* and MW, the chondrocytes were lysed with mammalian cell lysis buffer containing protease inhibitor cocktails and centrifuged at 15 000 ×g for 15 minutes, followed by determination of protein concentration in supernatants. The lysates were separated using a 12% SDS-PAGE gel under reducing conditions using 100 V for 1 hour. They were then transferred onto a PVDF membrane and blocked for 2 hours with 5% nonfat dry milk. The membranes were incubated overnight at 4°C with the desired primary antibodies: calpain, Bax, Bcl-2, cytochrome c, caspase-3, collagen II, aggrecan, and *β*-actin (at a dilution of 1 : 1000). They were then incubated with the appropriate HRP-conjugated secondary antibody. Lastly, enhanced chemiluminescence detection was performed.

### 2.14. Statistical Analysis

Each experiment was independently performed 3 times. SPSS Statistics 16.0 software was used for data analysis, and the data are shown as mean ± SD. The statistical analysis between groups was carried out using Student's* t*-test, and *P* < 0.05 was considered to be statistically significant.

## 3. Results

### 3.1. Identification of Chondrocytes

**Figure 1:**
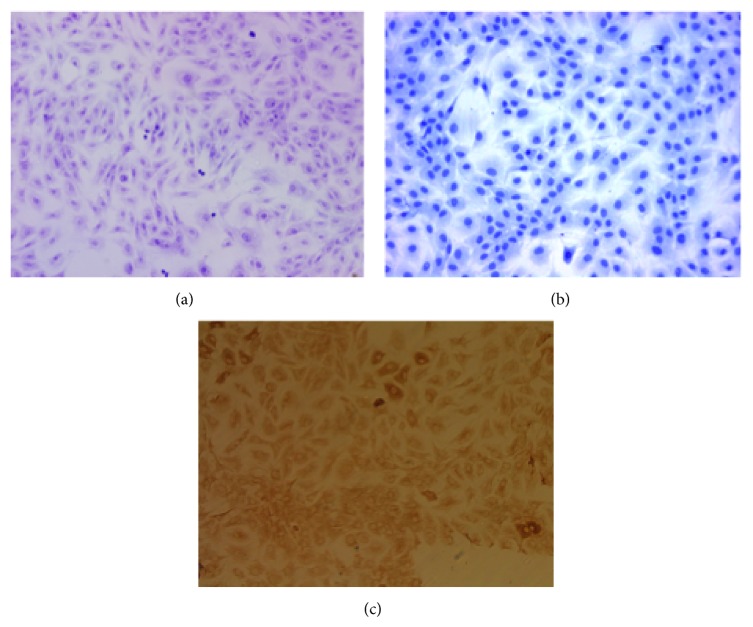
Identification of third generation of chondrocytes (×200). (a) Chondrocytes were stained using toluidine blue. Immunohistochemical staining of type II collagen demonstrated that (b) the cytoplasm of the negative group of chondrocytes was unstained, whereas (c) the cytoplasm of chondrocytes was stained.

To characterize the isolated chondrocytes, cells were identified using toluidine blue staining and* in situ* hybridization for type II collagen. Toluidine blue staining induced the formation of purple metachromatic granules within and around the cells. The nuclei were dark blue and round or oval-shaped (Figure 1(a)). The chondrocytes were evenly distributed with a clear polygonal-shaped border, and the cytoplasm was stained brown-yellow; however, the nuclear area was not stained (Figure 1(c)). In the negative control group, the cytoplasm of the chondrocytes was not stained brown-yellow (Figure 1(b)).

### 3.2. Morphological Observation of Chondrocytes

**Figure 2:**
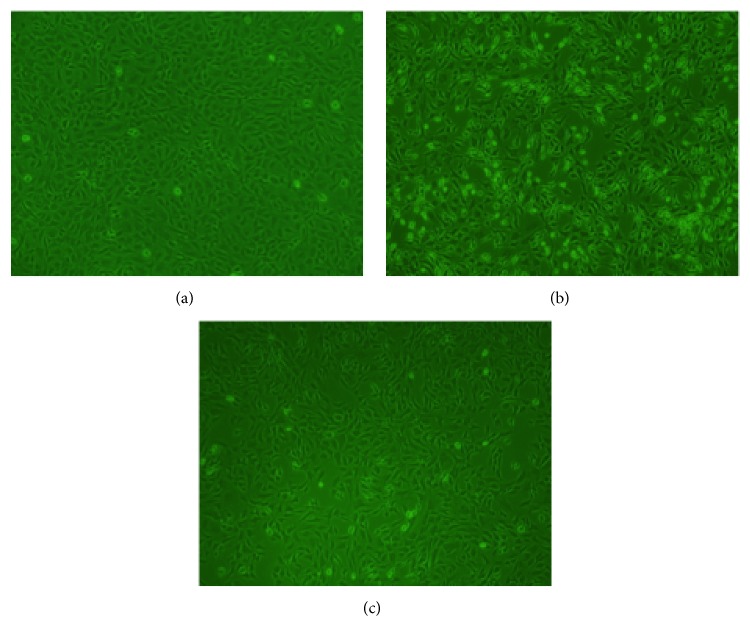
Effect of millimeter wave on the morphological changes of chondrocytes (×200). (a) Untreated control chondrocytes; (b) chondrocytes treated with 10 ng/mL TNF-*α* for 8 h. (c) Chondrocytes treated with 10 ng/mL TNF-*α* for 8 h and then treated with millimeter wave for 30 min. The morphological changes of chondrocytes were observed using phase contrast microscopy.

As observed under the microscope, the cell morphology of millimeter wave group and the blank control group was similar, resembling a pebble shaped growth (Figures 2(a) and 2(b)). The cell morphology changed considerably in the model group, as the cells exhibited increased polygonal shape, pseudopodia lengthened, some cells were shrunken, gaps between cells enlarged, and there was a decrease in the number of cells overall (Figure 2(c)).

### 3.3. Effect of MW on the TNF-*α*-Induced Apoptosis of Chondrocytes

**Figure 3:**
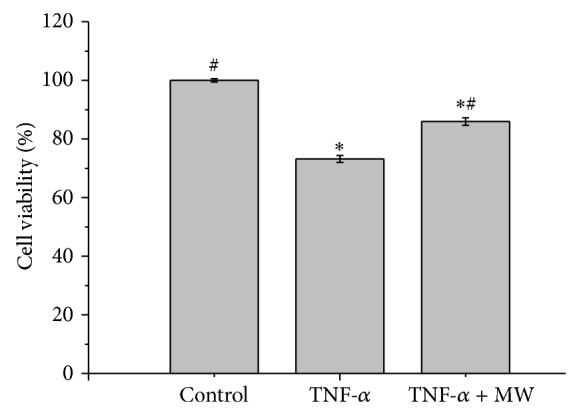
Effect of TNF-*α* on chondrocyte viability. Chondrocytes were treated with 10 ng/mL TNF-*α* or TNF-*α* and millimeter wave (MW) for 4 hours. Cell viability was determined by the MTT assay. Data are averages with SD (error bars) from three independent experiments. *P* < 0.05, compared with the untreated control. *P* < 0.05, compared with the 10 ng/mL TNF-*α* group.

As previously described, we used 20 ng/mL TNF-*α* for 4 hours to induce TNF-*α*-induced apoptosis in chondrocytes [13]. The effect of MW on chondrocyte viability was determined by MTT assay. As shown in Figure 3, 4 hours of treatment with 20 ng/mL of TNF-*α* reduced cell viability by 26.81% compared with the untreated control cells (*P* < 0.01). Four hours of treatment with 20 ng/mL of combined TNF-*α* and MW reduced cell viability by 14.04% compared with control cells. To determine the apoptotic rate of the chondrocytes treated with 20 ng/mL TNF-*α* and/or MW, we performed a DAPI assay. The untreated chondrocytes did not exhibit apoptosis, whereas the TNF-*α*-treated cells exhibited varying degrees of apoptosis. Most of the untreated cells had light blue round or oval nuclei, whereas the apoptotic cells had bright blue round or oval nuclei, indicating nuclear condensation. The apoptotic rate of the chondrocytes treated with 20 ng/mL TNF-*α* for 4 hours was significantly higher than those of the untreated cells (*P* < 0.01) or the cells treated with 20 ng/mL TNF-*α* and MW (*P* < 0.01) (Figure 5(a)). Chondrocyte apoptosis was determined using Annexin-V/PI staining followed by FCM analysis. As shown in Figures 5(b) and 5(c), the percentage of cells undergoing apoptosis was 2.87 ± 0.64%, 29.31 ± 3.28%, and 13.23 ± 4.00% in the control, TNF-*α*, and TNF-*α* + MW groups, respectively (early apoptosis found within the lower right [LR] and late apoptosis in the upper right [UR] quadrants in the FACS diagram). The 10 ng/mL TNF-*α* group showed significantly more apoptosis than the control group (*P* < 0.01). The TNF-*α* + MW group showed significantly less apoptosis compared with the TNF-*α* group (*P* < 0.01) and showed more apoptosis compared with the untreated control (*P* < 0.01) (Figures 5(b) and 5(c)).

### 3.4. Effect of MW on [Ca^2+^]_*i*_ in Chondrocytes

**Figure 4:**
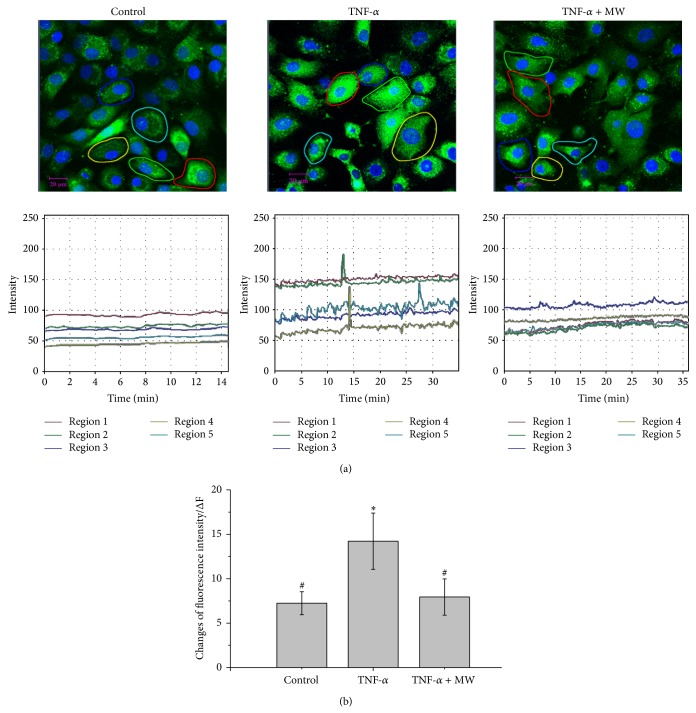
Effects of MW on intracellular [Ca^2+^]_*i*_ expression in chondrocytes. (a) Fluorescence of intracellular [Ca^2+^]_*i*_ was visualized by staining the chondrocytes with fluo-3/AM (green) and then counterstaining with Hoechst 33258 (blue). Images were captured under a confocal fluorescence microscope with a magnification of 400x. Five cells for each visual field were observed and the quantitative changes rule curve of intracellular [Ca^2+^]_*i*_ fluorescence, which changed over time, was analyzed. (b) The changes in intensity of fluorescence of intracellular [Ca^2+^]_*i*_. Colorful curve representing the intracellular calcium concentration corresponds to the cell circled by line with same color. Data are averages with SD (error bars) from three independent experiments. ^*^ *P* < 0.05, compared with the untreated control. ^#^ *P* < 0.05, compared with the 10 ng/mL TNF-*α* group.

**Figure 5:**
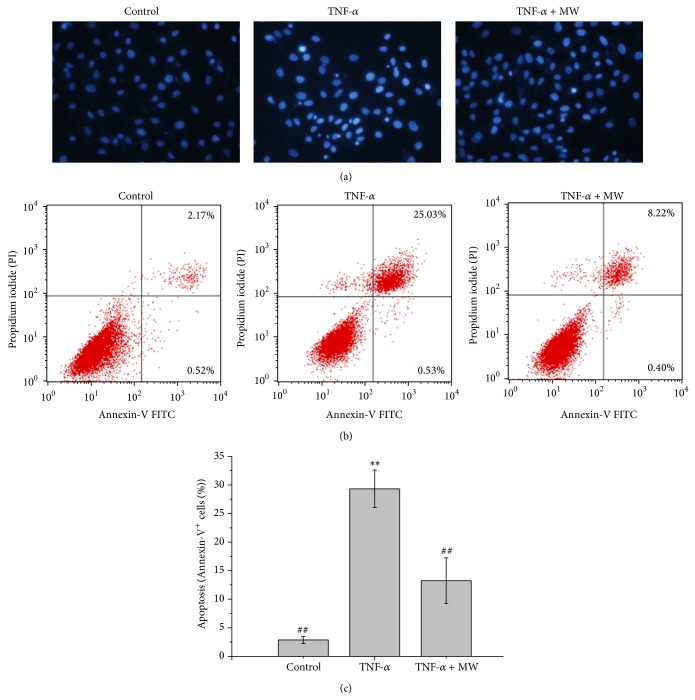
Effect of MW on cell apoptosis in chondrocytes. Chondrocytes were treated with 10 ng/mL TNF-*α* or TNF-*α* and MW, collected and stained with Hoechst 33258, and observed under a fluorescence microscope using Annexin-V^+^/PI and flow cytometry (FCM) analysis. (a) Visible condensed nuclei of chondrocytes stained with Hoechst 33258. (b) Apoptosis of chondrocytes analyzed by FACS. Images are representative of three independent experiments. (c) Quantification of FACS analysis. Data are averages with SD (error bars) from three independent experiments. ^*^ *P* < 0.05, compared with the untreated control. ^#^ *P* < 0.05, compared with the 10 ng/mL TNF-*α* group.

To further explore the change of TNF-*α*-induced apoptosis, we used the Ca^2+^ indicator fluo-3/AM to detect [Ca^2+^]_*i*_ changes after TNF-*α* and TNF-*α* + MW treatment. As shown in Figures 4(a) and 4(b), the [Ca^2+^]_*i*_ fluorescence intensity in the group treated with 20 ng/mL TNF-*α* (14.22 ± 3.17) was higher than in the control group (7.24 ± 1.30) (*P* < 0.05). The change of [Ca^2+^]_*i*_ in chondrocytes under TNF-*α* + MW treatment increased by a much smaller amount: 7.94 ± 2.04. There was no significant difference in intracellular calcium in the group treated with MW compared to the control. These results suggest that MW can prevent an influx of [Ca^2+^]_*i*_ and may inhibit apoptosis, whereas TNF-*α* alone can induce a calcium overload.

### 3.5. Effect of MW on the Loss of Mitochondrial Potential (ΔΨ)

The mitochondria-dependent pathway is the most common apoptotic pathway in vertebrate animal cells. Mitochondrial membrane permeabilization, accompanied by the collapse of electrochemical gradient across the mitochondrial membrane, is one of the key events of cellular apoptosis.

**Figure 6:**
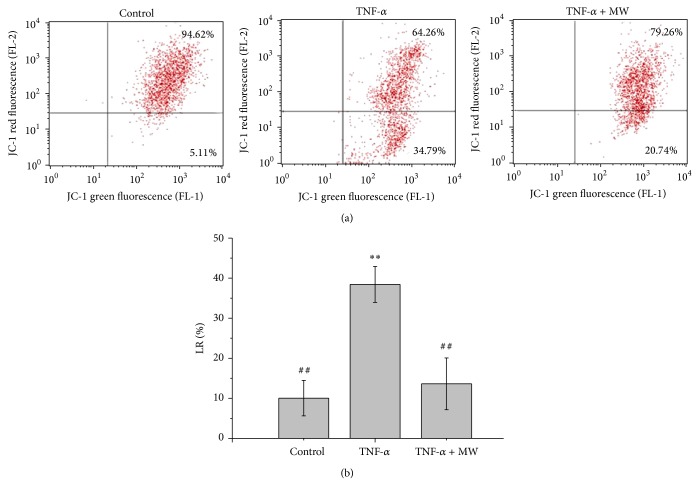
Effect of MW on the loss of mitochondrial membrane potential in chondrocytes. Chondrocytes were treated with 10 ng/mL TNF-*α* or TNF-*α* and MW and stained with JC-1. (a) The mean JC-1 fluorescence intensity was detected using FACS analysis. Data shown are representative of three independent experiments. (b) Quantification of FACS analysis. Data are averages with SD (error bars) from three independent experiments. ^*^ *P* < 0.05, compared with the untreated control. ^#^ *P* < 0.05, compared with the 10 ng/mL TNF-*α* group.

To investigate the mechanism of how MW induces apoptosis of chondrocytes, we examined the change in mitochondrial membrane potential after MW treatment using FACS analysis with JC-1 staining. JC-1 is a lipophilic, cationic dye that selectively enters into mitochondria. In unhealthy cells with high mitochondrial potential, JC-1 forms J-aggregates with intense red fluorescence (590 nm). Conversely, in apoptosis, the mitochondrial membrane potential collapses so that the JC-1 does not accumulate within the mitochondria but remains in the cytoplasm in monomeric form, emitting green fluorescence (525 nm). These differences in fluorescence can be detected with FACS analysis using green and red JC-1 channels. As shown in Figure 6(a), the JC-1 fluorescence shifted from a JC-1-green-bright/JC-1-red-bright signal in the untreated chondrocytes to a JC-1-green-bright/JC-1-red-dim signal in the cells treated with TNF-*α*, indicating a TNF-*α*-induced loss of mitochondrial membrane potential in the chondrocytes. The chondrocytes treated with TNF-*α* + MW exhibited a JC-1-green-bright/JC-1-red-bright signal. As shown in Figure 6(b), the percentages of apoptotic cells (LR quadrant) in the control, 10 ng/mL TNF-*α*, and TNF-*α* + MW groups were 10.04 ± 4.42, 38.43 ± 4.48, and 15.99 ± 6.15, respectively. Compared with the control group, the 10 ng/mL TNF-*α* group had a greater number of cells with low red fluorescence (FL-2 [LR]) (*P* < 0.01), indicative of a reduction in the ΔΨ and of the induction of apoptosis. The TNF-*α* + MW group had significantly fewer cells with low red fluorescence (FL-2 [LR]) (*P* < 0.01) as compared to the TNF-*α* group. This indicates that MW treatment inhibited TNF-*α*-induced apoptosis.

### 3.6. Effect of MW on the Protein Expressions of Calpain, Bax, Bcl-2, Cytochrome c, Caspase-3, Collage II, and Aggrecan

**Figure 7:**
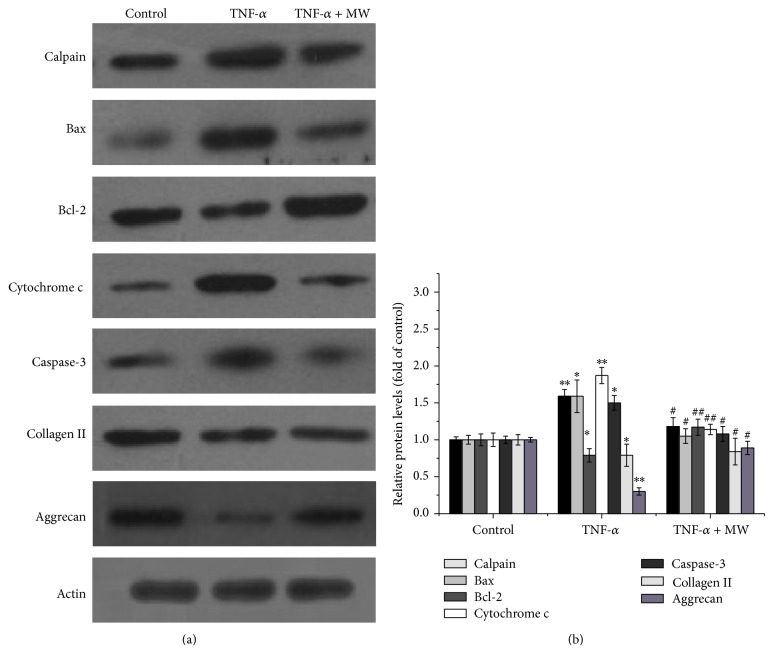
Effect of MW on the expression of calpain, Bax, Bcl-2, cytochrome c, caspase-3, collagen II, and aggrecan in chondrocytes. (a) The protein expression levels of calpain, Bax, Bcl-2, cytochrome c, caspase-3, collagen II, and aggrecan were analyzed by Western blotting. *β*-actin was used as the internal control. (b) Semiquantification of protein blots shown in (a). Data are averages with SD (error bars) from three independent experiments. ^*^ *P* < 0.05; ^**^ *P* < 0.01, compared with the untreated control. ^#^ *P* < 0.05, ^##^ *P* < 0.01, compared with the 10 ng/mL TNF-*α* group.

Bcl-2 family proteins, including antiapoptotic members such as Bcl-2 and proapoptotic members such as Bax, are key regulators of mitochondria-mediated apoptosis. Upon apoptosis induction, the proapoptotic protein Bax is translocated from the cytosol to the mitochondria, where it promotes release of cytochrome c, a caspase-activating protein. Calpain-mediated Bax cleavage activity was found in the mitochondrial membrane-enriched fraction. Data from a Western blot analysis showed that the levels of protein expression of calpain, Bax, cytochrome c, and caspase-3 were profoundly reduced with MW treatment, while the pattern of protein expression of Bcl-2, collagen II, and aggrecan was increased (Figures 7(a) and 7(b)).

## 4. Discussion

Osteoarthritis (OA) is a multifactorial disease characterized by the breakdown of hyaline articular cartilage and the formation of osteophytes. The chondrocyte is the only cell type present in mature cartilage that is responsible for extracellular signals and the maintenance of cartilage homeostasis. Therefore, the functional changes of chondrocytes play an important role in OA, as they contribute to the degradation of articular cartilage and thus to the pathogenesis of OA [22, 23]. We previously demonstrated that chondrocytes are obtained by mechanical and chemical isolation methods and that the purity of the isolated chondrocytes is high [12, 24]. This process allows for the investigation of the effect of MW on chondrocyte apoptosis. Several studies have reported that osteoarthritic chondrocytes have a very low proliferative activity; therefore, inhibiting chondrocyte apoptosis may be an efficient treatment strategy in the cure or delay of the progression of OA.

One of the key inflammatory factors responsible for articular cartilage degeneration, TNF-*α*, is involved in the synovial inflammatory response, the initiation of chondrocytic apoptosis, the imbalance between chondral damage and repairs, and the eventual acceleration of articular cartilage degeneration [25]. We found that a 10 ng/mL TNF-*α* treatment for 8 hours was optimal for the induction of chondrocyte apoptosis, and this dose was used for further studies [13].

TNF-*α* caused an increase in the production of [Ca^2+^]_*i*_ and a downregulation of mitochondrial membrane potential (ΔΨ*m*), resulting in apoptosis. These findings suggest that Ca^2+^ potentially plays a role in TNF-*α*-induced apoptosis in chondrocytes. Ca^2+^ is a factor in excitation-contraction coupling, and the increase of [Ca^2+^]_*i*_ is dependent on extracellular Ca^2+^ entry and Ca^2+^ release from intracellular stores such as sarcoplasmic reticulum (SR) or mitochondria. Precise control of intracellular Ca^2+^ homeostasis is vital for the regulation of a chondrocyte's functions. The involvement of a Ca^2+^ influx has been reported for various apoptotic stimuli. In tributyltin-induced T-cell apoptosis, a rise in Ca^2+^ concentration is required for postmitochondrial events, namely, the activation of caspases [26]. Therefore, the rise of intracellular Ca^2+^ is a hallmark of apoptosis induction in multiple paradigms.

The accumulated data suggest that the mitochondria-initiated death pathway plays an important role in triggering apoptosis in response to those stimuli. In the mitochondria-initiated death pathway, mitochondria undergoing permeability transition release apoptogenic proteins such as cytochrome c or apoptosis-inducing factors from the mitochondrial intermembrane space into the cytosol. Released cytochrome c can activate caspase-9, which in turn cleaves and activates executioner caspase-3 [27].

Millimeter wave treatment has been used as a nonmedicinal, noninvasive physical therapeutic regimen for the treatment of osteoarthritis. The frequency of the millimeter wave (0.5 × 10^10^ to 3 × 10^12^) is in the same range of the frequency of coherent oscillation produced by biological organisms during metabolism. Therefore, the energy of the millimeter wave can be absorbed by the organism through resonance. The energy can then be transferred so that it interferes with signal transduction in the organism, dynamically regulating metabolism. We treated TNF-*α*-induced chondrocytes with millimeter wave therapy with the aim of further investigating the potential mechanisms of action through which millimeter wave treatment regulates chondrocytic functions.

Our results show that an 8-hour treatment of TNF-*α* increases the level of intracellular [Ca^2+^]_*i*_ and results in chondrocytic apoptosis. Conversely, millimeter wave treatment efficiently suppresses apoptosis through the downregulation of intracellular [Ca^2+^]_*i*_, reduces cell membrane permeability and increases ΔΨ*m*, and inhibits the activation of calpain, which in turn mediates Bax cleavage and cytochrome c release and initiates apoptotic execution.
